# Direct Calculation of Electron Transfer Rates with the Binless Dynamic Histogram Analysis Method

**DOI:** 10.1021/acs.jpclett.3c02624

**Published:** 2023-10-30

**Authors:** Zsuzsanna Koczor-Benda, Teodora Mateeva, Edina Rosta

**Affiliations:** † Department of Physics and Astronomy, University College London, London WC1E 6BT, United Kingdom; ‡ Department of Chemistry, University of Warwick, Coventry CV4 7AL, United Kingdom; § Department of Physics, King’s College London, London WC2R 2LS, United Kingdom

## Abstract

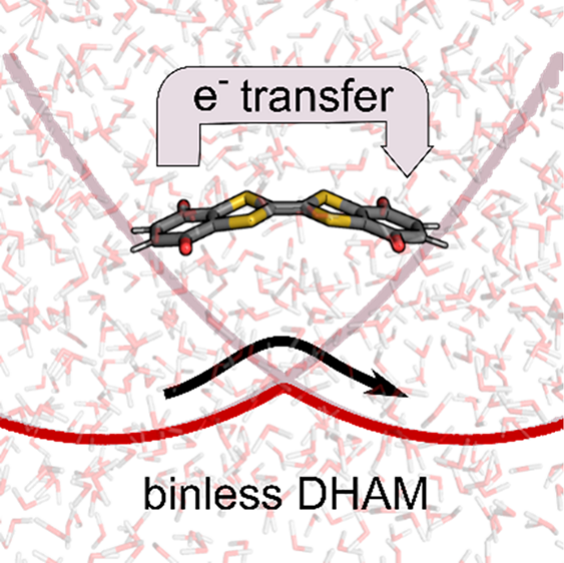

Umbrella sampling molecular dynamics simulations are widely used to enhance sampling along the reaction coordinates of chemical reactions. The effect of the artificial bias can be removed using methods such as the dynamic weighted histogram analysis method (DHAM), which in addition to the global free energy profile also provides kinetic information about barrier-crossing rates directly from the Markov matrix. Here we present a binless formulation of DHAM that extends DHAM to high-dimensional and Hamiltonian-based biasing to allow the study of electron transfer (ET) processes, for which enhanced sampling is usually not possible based on simple geometric grounds. We show the capabilities of binless DHAM on examples such as aqueous ferrous-ferric ET and intramolecular ET in the radical anion of benzoquinone–tetrathiafulvalene–benzoquinone (Q-TTF-Q)^−^. From classical Hamiltonian-based umbrella sampling simulations and electronic coupling values from quantum chemistry calculations, binless DHAM provides ET rates for adiabatic and nonadiabatic ET reactions alike in excellent agreement with experimental results.

The calculation of free energy profiles is central for modeling chemical reactions. In molecular dynamics (MD) simulations, it is common to employ biasing functions to facilitate the exploration of otherwise rarely visited regions of the free energy surface. To overcome barriers in free energy surfaces, the umbrella sampling (US) method and analogous biased simulations are often used, where the free energy profile is estimated along one or more collective variables (CVs).^1,2^ In chemical reactions, there is usually one or a small number of nuclear coordinate changes that describe the transition from reactants to products. In electron transfer (ET) reactions that are not coupled to other chemical changes (e.g., proton transfer), this is not the case. In outer-sphere ET reactions, for example, the reactants and products are different only in the rearrangement of the electron density and the corresponding complex changes in the environment. For ET reactions, instead of nuclear coordinates, the reaction coordinate is better defined as the energy gap (i.e., difference) between diabatic charge localized states.^3,4^

To unbias US-type enhanced sampling simulations and to construct the free energy profile along one (or a few) reaction coordinate(s), the weighted histogram analysis method (WHAM)^5^ is commonly used. However, WHAM disregards the time sequence information within simulation trajectories and therefore kinetic information is lost. To obtain molecular kinetics, the dynamic histogram analysis method (DHAM) was developed,^6^ as well as its more robust implementation using rate matrices instead of transition matrices via DHAMed.^7^ However, when simulations are biased along many coordinates or via biasing functions that may not be related to the reaction coordinate, DHAM needs to be reformulated. We introduce here a modification of DHAM, called binless DHAM, that approximates the unbiased Markov matrix and thus allows for unbiasing in such cases. The term “binless” is used to reflect the similarity to the multistate Bennett acceptance ratio estimator (MBAR),^8^ which is an analogous binless implementation of WHAM.^9^ The key advantage of binless DHAM over MBAR is that it also directly provides reaction rates. This provides an alternative to Eyring’s transition state theory (TST) or Marcus theory for nonadiabatic ET, which calculates rates from activation free energies or Marcus parameters (driving force, reorganization energy, and electronic coupling), respectively. Another method that reports being able to obtain rates directly is dTRAM.^10^ Similarly to DHAM, dTRAM does not require data to be sampled from global equilibrium and provides maximum-likelihood estimates of stationary quantities. However, no rates have been reported to be calculated for model systems. While dTRAM in principle can also provide kinetics from multiensemble simulations, this requires that unbiased simulation data are also included, which is typically not available for ET simulations and in many other cases.^11^

We demonstrate the binless DHAM method on various systems, focusing on condensed-phase ET reactions, where a dynamical description of the solvent is essential. To sample different ensembles of configurations and build diabatic free-energy surfaces, we perform Hamiltonian-based US MD, where we vary the charges of donor and acceptor subunits incrementally. This US technique for ET processes has been previously applied in semiclassical and ab initio MD studies^12−14^ and is also similar to λ-dynamics^15^ used in the context of protein–ligand binding.

Our first example is the ferrous-ferric self-exchange ET process, an often-used test case for new methodologies and an example for nonadiabatic ET. Previous molecular dynamics simulations of this system used classical force fields,^13,16^ ab initio MD (Car–Parrinello, CPMD),^14^ or quantum mechanics/molecular mechanics (QM/MM) MD^17^ to determine free energy profiles and Marcus parameters. The electronic coupling has been investigated with various quantum chemistry methods such as fractional occupation number density functional theory (FON-DFT),^18^ restricted open-shell Hartree–Fock ROHF,^19^ and a model considering a quantal electron and classical Fe^3+^ ions.^13^ Here we use frozen density embedding (FDE)^20,21^ to calculate the electronic coupling on frames from MD simulations.

The second example is the intramolecular electron transfer (IET) within the radical anion of the benzoquinone–tetrathiafulvalene–benzoquinone triad (Q-TTF-Q)^−^ in four different solvents: *tert*-butyl alcohol (tBOH), dichloromethane (DCM), ethyl acetate (ETA), and water. The (Q-TTF-Q)^−^ anion is a type II compound according to the Robin-Day classification scheme,^22^ in polar solvents, meaning that its ground state is charge-localized and ET between the two parts of the molecule is well approximated by the adiabatic mechanism. Organic compounds capable of IET, such as tetrathiafulvalene (TTF) derivatives, are gathering interest for their potential as organic conductors.^23^ The understanding of the IET in the (Q-TTF-Q)^−^ anion and other TTF derivatives could enable the engineering of the ET process which has potential applications in the design of molecular wires and other applications in nanotechnology.^24,25^ However, the estimation of the IET currently represents a challenging task for computational methods.^26^ The correct description of the system poses a challenge for quantum chemistry methods.^27−34^ The electronic coupling was previously calculated with CDFT in the gas phase,^27^ with CDFT on ab initio MD simulation frames for the unconstrained charge delocalized state including explicit solvent,^28^ directly with CDFT MD,^35^ and with time-dependent (TD) DFT.^29^ We add to this variety of techniques by determining the coupling with an equation-of-motion coupled cluster (EOM-CC) approach as well.

For the ferrous-ferric ET, we achieve excellent agreement with the experimental rate (5.2 × 10^2^ s^–1^ calculated vs 7.9 ×10^2^ s^–1^ experimental^36^). For the IET in (Q-TTF-Q)^−^, the calculated rates are within one order of magnitude of the experimentally reported ones.

The relation between biased and unbiased Markov transition probability matrices *M* can be expressed by solving the Smoluchowski diffusion equation^37^ for transition probabilities *p*(*i* → *j,τ*) from state *i* to *j* within a lag time τ as follows:
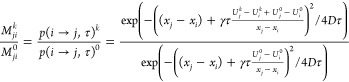
1with superscipt *k* denoting biased simulation *k* and 0 denoting the unbiased case. DHAM assumes a shared diffusion coefficient *D* between biased and unbiased simulations, which can nevertheless be position-dependent. *U* is the potential energy along the *x* reaction coordinate, and γ = *D*/*k*_*B*_*T* is the mobility of the system. Expanding the squared terms in eq 1 and omitting all τ^2^ terms lead to the square root approximation at short lag times,^38^
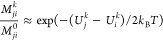
2In regular DHAM,^39^ the unnormalized Markov matrix is defined as
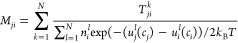
3where data is binned along *x*, and 
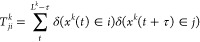
 gives the transition count from bin *i* to bin *j* in simulation window *k*, with data saved and analyzed at the frequency of τ (lag time) from the overall *L*^*k*^ length of simulation *k*.  is the number of transitions initiating from bin *i*. The bias *u*_*i*_^*l*^ = *U*_*i*_^*l*^ – *U*_*i*_^0^ is evaluated at each bin center *c*_*i*_, assuming that the biasing is also done along *x*.

The formally exact expression in eq 3 can be approximated by calculating the bias at the actual value of the reaction coordinate for each frame (*x*_*t*_^*k*^) instead of *c*_*i*_, similarly to the binless formulation of WHAM.^9^ This approximation becomes exact in the limit of very small bin sizes. With this binless formulation, it is then straightforward to obtain *M*_*ji*_ for any bias along arbitrary coordinates *q*_*t*_^*k*^
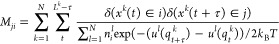
4Equation 3 can also be approximated by evaluating *u*^*l*^(*q*_*t*_^*k*^ ∈ *i*) for all *q*_*t*_^*k*^ data points that fall into bin *i* and using the average or median values in the denominator (see section S1 of the Supporting Information). This was also used to re-weight free energies in a binless form of the conformational states for the Ala5 peptide with DHAMed.^40^

After normalizing the columns of *M*_*ji*_, its right eigenvector corresponding to eigenvalue 1 gives the normalized equilibrium probabilities *p*_*i*_, from which the free energy profile is calculated as *G*_*i*_ = −*k*_B_*T*ln *p*_*i*_. In the ET examples below, we calculate the biasing energy with respect to the adiabatic ground state energy.
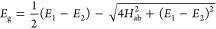
5Here *E*_1,2_ are the charge localized diabatic states and *H*_ab_ is the electronic coupling between them.

Within semiclassical TST the ET rate can be written^41^ as
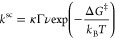
6where Δ*G*^‡^ is the activation free energy, ν is the nuclear frequency factor that gives the frequency of reaching the transition state (TS), and κ is the electronic transmission coefficient that describes the probability of electron transfer at the transition state. Γ is the quantum correction factor accounting for nuclear quantum effects such as nuclear tunneling that can enhance the reaction rate, and it is usually considered to be 1; thus, we leave it out from the following formulas to be consistent with previous studies. The magnitude of κ depends on the electronic interaction between the redox pairs; when their interaction is sufficiently strong, then κ ≈ 1 and the reaction is labeled as adiabatic, and when their coupling is small then κ < 1 and the reaction is nonadiabatic.

In contrast, in binless DHAM the reaction rate (*k*^M^) is calculated directly from the second largest eigenvalue (*m*) of the normalized *M*_*ji*_:
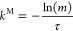
7The rate calculated this way is equivalent to the adiabatic rate from TST (eq 6, κ = 1 case), providing a new way to determine the pre-exponential factor ν as
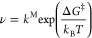
8This can be compared to the common approximation of ν as *k*_B_*T/h* or as the frequency of the vibrational mode transforming reactants to products (when such mode can be identified). To access nonadiabatic rates as well, only a correction by κ is needed, which can be calculated from Landau–Zener theory.^42−44^
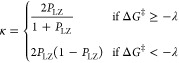
9.1
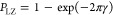
9.2
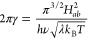
9.3Since the reorganization energy λ can be determined from the diabatic free energy profiles, the only external parameter needed to determine the nonadiabatic rate *κk*^M^ through eqs 7 and 9.1—9.3 is *H*_ab_, which is already required to build the adiabatic ground state (eq 5).

In the Condon approximation,^45,46^*H*_ab_ is constant along the reaction coordinate, and its value is half the energy gap of the two adiabatic potential energy surfaces at the transition state. Calculating *H*_ab_ directly from the excitation energy is usually not reliable with single reference methods, which break near degeneracies of the ground and excited states. However, to ensure a balanced description of the interacting states,^47^ one can take a well-behaved state such as the ground state of the neutral Q-TTF-Q as a starting point and use the electron attachment variant of equation-of-motion coupled cluster theory (EOM-EA-CC)^48^ to get the ground and first excited states of the radical anion (Q-TTF-Q)^−^.

A disadvantage of EOM-CC methods is that the solvent can be considered only implicitly due to the high computational cost. Explicit consideration of solvent is possible with DFT methods; however, DFT functionals are prone to electron delocalization error,^49^ giving an overstabilized adiabatic state and thus overestimating the coupling.^27,29^ Instead, the electronic coupling is better calculated with constrained density functional theory (CDFT)^27^ or frozen density embedding (FDE),^20,21^ which have a smaller delocalization error due to using only localized diabatic states. However, these methods are not always reliable either, or in some cases *H*_ab_ can be particularly sensitive to the fraction of exact exchange in the functional, e.g., CDFT-CI is known to give erroneous couplings for the ferrous-ferric ET reaction due to fractional charge transfer.^50^

## Methods

Details of the Monte Carlo simulations for the 1D two-state analytical potential are given in section S2. For ferrous-ferric ET, charges and van der Waals radii were interpolated between the reactant (Fe^2+^–Fe^3+^) and product (Fe^3+^–Fe^2+^) for 11 simulation windows. For (Q-TTF-Q)^−^, reactant and TS structures were optimized at the B3LYP/TZVP level using the CPCM implicit water model with Gaussian 09.^51^ CHELPG atomic charges for the two structures (Table S1) were linearly interpolated to set up a total of four simulation windows. Classical MD simulations with Amber force field^52^ and TIP3P water model^53,54^ were run for 2.5 (ferrous-ferric ET) and 2 ns (IET in (Q-TTF-Q)^−^), respectively, with 2 fs step size. Longer simulations were run in the organic solvents to ensure the proper equilibration of the systems. For further details see the sections S2 and S3 of the SI. For ferrous-ferric ET, the electronic coupling was calculated with FDE for 10 MD frames near the TS including only the first solvation shell. Calculations were run with PBE functional, TZP basis set, and PW91k for the nonadditive kinetic energy using the ADF software.^55^ For (Q-TTF-Q)^−^, the electronic coupling was calculated at the B3LYP/TZVP TS structure using the back-transformed PNO-based EOM-EA-CCSD method^56^ available in ORCA^57^ with the CPCM implicit water model, aug-cc-pVTZ basis set, and corresponding auxiliary bases.

First, we apply binless DHAM to simple umbrella sampling simulations for two examples, namely (i) a model potential and (ii) Na^+^ passage through an ion channel, to test how it compares to regular DHAM and WHAM methods. We then present applications that are beyond reach for these methods: ferrous-ferric ET and IET in (Q-TTF-Q)^−^. We compare free energy profiles to MBAR results in these cases, and present rates calculated directly from the Markov matrix. The results are then compared to experimental ET rates and Marcus parameters determined in previous works.

**Figure 1:**
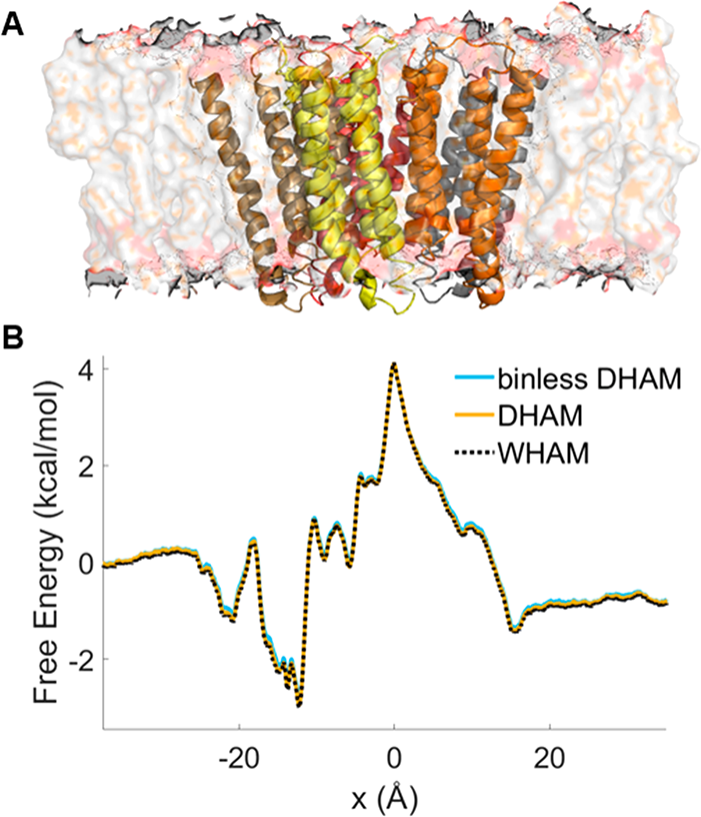
Reconstruction of the free energy profile from umbrella sampling simulations for Na^+^ ion passage through the GLIC ion channel. (A) Unit cell of the simulation system. The five transmembrane units of GLIC are shown in different colors, as per the original depiction in ref (58). (B) Binless DHAM (blue) and DHAM (orange) profiles are plotted against the WHAM profile (black dashed lines) obtained by Zhu and Hummer.^58^

Binless DHAM reconstructs the exact free energy profile successfully for the 1-D model potential, giving a profile closely matching the regular DHAM (Figure S1). For the passage of Na^+^ ions through the transmembrane pore of the GLIC channel (Figure 1A, simulations by Zhu and Hummer^58^), binless DHAM results are in very good agreement with both DHAM and WHAM results (Figure 1B) using a lag time of 100 fs and bin number of 1000. Our tests show that the convergence of the profile with respect to bin size and lag time needs to be verified in each case^59^ (Figures S2–S3). For DHAM, and Markov state model-based methods in general, smaller bin sizes provide more accurate results, as the diffusion process is closer approximated with better discretization.^60,61^

**Figure 2:**
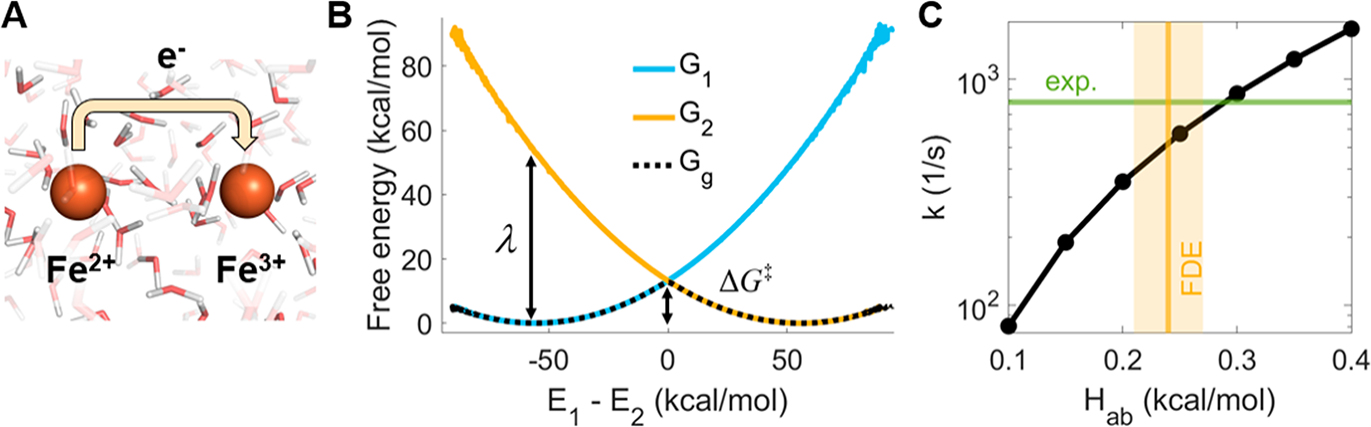
(A) Depiction of the ferrous-ferric electron transfer reaction in water. (B) Binless DHAM free energy profiles of diabatic states (*G*_1,2_) and the adiabatic ground state (*G*_g_). The reorganization energy λ and the activation free energy Δ*G*^‡^ are also shown. (C) ET rates calculated using binless DHAM as a function of *H*_ab_. The experimental rate^36^ is shown in green, while *H*_ab_ values calculated with FDE (mean and standard error) are shown in yellow.

For the ferrous-ferric ET reaction (Figure 2A), the diabatic and adiabatic free energy profiles unbiased with binless DHAM are shown in Figure 2B. For unbiasing high-energy states such as the diabatic states, high numerical precision is needed.^6^ We also tested the alternative approach using the mean bias (eq S1), but we only see a difference in performance for a significantly reduced number of data points, where it performs slightly worse than eq 4 (see Figure S4). Binless DHAM gives a very similar free energy profile to MBAR (Figure S5), and the diabatic states are well approximated by a quadratic function (Figure S6), in line with Marcus theory. The reorganization energy λ is calculated from the fitted curves to be 53.1 kcal/mol (see section S8), which is only slightly higher than the experimental 48.4 kcal/mol.^36,62^ In contrast, other classical MD simulations significantly overestimate λ, giving about 83 kcal/mol.^13,16^ Our improved estimate of λ is probably due to varying the van der Waals radii between Fe^2+^ and Fe^3+^. Quantum chemical description of the system was shown to provide even more accurate λ; DFT with the four-point approach^63^ gives 48.7 kcal/mol,^36^ while CPMD with a penalty function spin-polarized DFT approach gives 46 kcal/mol.^14^

FDE calculations on 10 snapshots from the simulation give *H*_ab_ = 0.24 ± 0.03 kcal/mol, which is in good agreement with values reported in the literature: 0.25 ± 0.06 kcal/mol with FON-DFT+U,^18^ 0.28 kcal/mol with ROHF,^19^ and 0.35 kcal/mol with a model considering an electron in the pseudopotential field of two classical Fe^3+^ ions.^13^ The ET rate as a function of *H*_ab_ is shown in Figure 2C. Our binless DHAM methodology with the mean FDE coupling yields a rate of 5.2 × 10^2^ s^–1^, in excellent agreement with the experimental ET rate 7.9 × 10^2^ s^–1^.^36,64^

The activation free energy Δ*G*^‡^ is 12.8 kcal/mol, somewhat higher than 11.3 kcal/mol with penalty function DFT.^14^ The nuclear frequency factor ν = 8.87 × 10^13^ s^–1^ is also higher than 1.16 × 10^13^ s^–1^ calculated in ref (36) from the symmetric Fe–O stretching frequency. In comparison, *k*_B_*T*/h is 6.32 × 10^12^ s^–1^ at a temperature of 303.15 K. Since κ is dependent on ν, it is not surprising that our calculated κ = 0.013 is also different from previously reported values 0.06–0.0679^36^ and 0.15;^19^ nevertheless, it is in line with the nonadiabatic nature of this reaction. The agreement with ref (36) is much improved if we look at the prefactors (κν) directly. We note that although the quantum correction factor Γ is often assumed to be 1, previous studies indicate that for this reaction it can be as high as 10–70,^65−67^ increasing the calculated rate, which would worsen the agreement with experimental rates.

**Figure 3:**
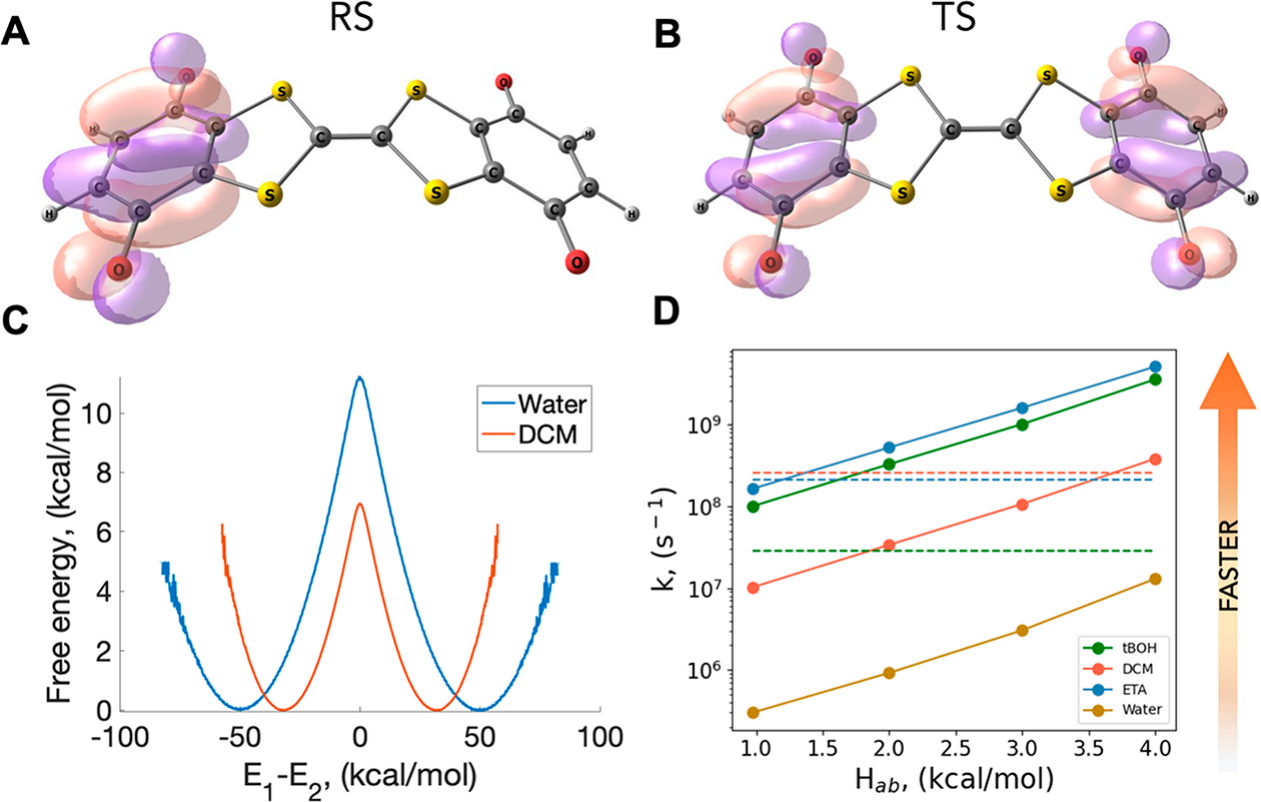
IET in (Q-TTF-Q)^−^. Dominant molecular orbitals describe electron attachment to neutral Q-TTF-Q to form (A) the reactant state (RS) and (B) the transition state (TS) of the (Q-TTF-Q)^−^ anion. Pink and purple colors represent the different phases of the wave function. (C) Binless DHAM free energy profiles plotted using *H*_*ab*_ coupling values from our EOM-CC calculations for water (blue line) and DCM (red line) as an example of the very different rates of ET. (D) Calculated ET rates as a function of *H*_ab_. Experimental rates for tBOH (green), DCM (red), and ETA (blue) are also shown as dashed lines.^68^

Both the RS and TS structures of (Q-TTF-Q)^−^ are nonplanar. The adiabatic ground state charge distribution is shown via the molecular orbitals occupied by the excess electron (Figure 3A and B for RS and TS, respectively), as calculated with EOM-EA-CCSD. From the energy difference of the adiabatic states at the TS, we obtain 1.0 kcal/mol coupling. In comparison, different CDFT-based approaches yielded an *H*_ab_ of 3.0 kcal/mol in gas phase,^27^ while with explicit water solvent *H*_ab_ is calculated as 4.2 kcal/mol^28^ (CDFT on frames from unconstrained MD) or 2.0 kcal/mol^35^ (CDFT MD with PBE0 functional). The excitation energy approach with TDDFT and D-COSMO-RS solvent model for 10:1 ethyl acetate/*tert*-butyl alcohol resulted in 2.0 kcal/mol coupling.^29^ As *H*_ab_ values are not unique, there is no standard method of determining these. Here, we compared calculated and experimental rates^68^ obtained with various choices of *H*_ab_ using different solvents.

We calculated the free energy profiles for the intramolecular electron transfer in four solvent environments: *tert*-butyl alcohol (tBOH), ethyl acetate (ETA), dichloromethane (DCM), and water. The binless DHAM free energy profiles for water and dichloromethane (DCM) are shown in Figure 3C. Binless DHAM has excellent agreement with MBAR in all cases (Figure S5). The energy barriers, the calculated rates (using a coupling of 0.97 kcal/mol), and the reorganization energies are summarized in Table 1, together with the experimental dielectric constants and the measured IET rates for all solvents except for water.^68^ Our calculations suggest that the process follows similar rates in tBOH, ETA and DCM, but it is considerably slower in water. Our predicted rates are within an order of magnitude of the experimental rates, using the *H*_ab_ values from around 1–3 (Figure 3D), which demonstrates a good agreement in general and shows that our method could be used to determine *H*_ab_ values if the rates are known or vice versa, that experimental rates can be determined if *H*_ab_ values are calculated. The dielectric constant, ε, is much higher for water than the rest of the organic solvents we modeled (Table 1), which also corresponds to the much slower rate we observe for the IET in water. The dielectric constants are broadly similar for the three organic solvents, as are the ET rates, within about an order of magnitude for both the calculated and experimental values (Table 1). We note that while the precise ordering correlates perfectly between the calculated rates and the reorganization energy λ, it does not perfectly correlate across the experimental rates and measured dielectric constants (Table 1). Experimentally, λ is estimated from a broad intervalence charge transfer band to be around 22 kcal/mol in 10:1 ethyl acetate/*tert*-butyl alcohol,^64^ which is also matched well by TDDFT predictions of 16.1 kcal/mol for the same solvent mixture.^24^ In line with the adiabatic classification of the reaction, the calculated κ is 1.00 for all solvent environments.

**Table 1:** Calculated Energy Barriers from the First Eigenvector of the Markov Matrix, Calculated Rates from the Second Eigenvalue of the Markov Matrix, Derived Pre-Exponential Factors and Reorganization Energies^a^ vs Experimentally Measured Rates for the Respective Solvents, and Measured Dielectric Constants (ε)

solvent	energy barrier (kcal/mol)	calculated rate (s^–1^)	pre-exponential factor (s^–1^)	reorganization energy (λ, kcal/mol)	experimental rate (s^–1^)^b^	dielectric constant ε^c^
tBOH	6.61	9.97 × 10^07^	5.77 × 10^12^	29.69	2.89 × 10^07^	10.9
ETA	5.91	1.69 × 10^08^	3.04 × 10^12^	26.97	2.10 × 10^08^	6.02
DCM	6.94	1.03 × 10^07^	1.04 × 10^12^	30.79	2.58 × 10^08^	8.93
Water	11.23	3.00 × 10^05^	3.73 × 10^13^	48.24	n/a	80.1

a Derived using a coupling of H_ab_ = 0.97 kcal/mol for the IET in four solvent environments.

b See ref (68).

c See ref (71).

Using the calculated barrier heights and the relaxation times from the eigenvalues of the unbiased Markov matrices, we also calculated the pre-exponential factor ν for the adiabatic rates in the form of eq 8. We have an excellent agreement with the standard kT/h values (6.32 × 10^12^ s^–1^ at 303.15 K, Table 1), demonstrating that our reaction coordinate correctly captures the rate limiting factors for this process. Using low dimensional reaction coordinates that miss key relevant degrees of freedom could result in too low free energy barriers, even if the sampling is perfect.^69^ This could result in an apparent pre-exponential factor that is significantly different from the kT/h value, as observed in, e.g., umbrella sampling MD simulations of small molecules membrane permeation (ν ∼ 10^8^).^70^ Analogously, using reaction coordinates that better capture the rate limiting process for the IET could increase the barrier heights in IET simulations and thus could result in better agreement with experimental rates without invoking changes in the nuclear tunneling effects.

We derived a binless formulation of the dynamic histogram analysis method that can be used to build the free energy profile from molecular dynamics simulations biased along many arbitrary coordinates, such as Hamiltonian-based biasing. It is especially suited for the investigation of electron transfer (ET) reactions, which we demonstrated on two examples, ferrous-ferric ET and IET in (Q-TTF-Q)^−^. With binless DHAM, reaction rates can be directly calculated from the Markov transition probability matrix, also providing an alternative route to determine the nuclear frequency factor of the transition state theory. The only external parameter needed to access adiabatic or nonadiabatic ET rates is the electronic coupling between redox pairs, readily calculated with frozen density embedding, constrained density functional theory, or excited state methods.

Our method gives nearly identical results to DHAM and WHAM on simulations biased along a low-dimensional reaction coordinate and to MBAR when biasing is along arbitrary coordinates, provided that the profile is converged with respect to bin size and lag time. Importantly, using the binless DHAM, the pre-exponential factor can be calculated from the unbiased Markov matrix estimate; hence, not only the free energy but also the kinetic rates are directly obtained from biased simulations.

Here, we demonstrate that using a binless DHAM for unbiasing ET simulations, the rates can be directly determined from MD simulations using different model Hamiltonians. Using appropriate coupling values, we obtained excellent agreement with experimental rates for both adiabatic and nonadiabatic ET reactions. We obtain IET rates within an order of magnitude of the experimental rates for (Q-TTF-Q)^−^ in three different organic solvents using our *H*_ab_ coupling value determined using EOM-CC. Additionally, our calculated reorganization energies are also in good agreement with experimental estimates. Apart from ET reactions, binless DHAM can also be potentially used to calculate kinetic rates in cases where different Hamiltonians are used for sampling and energy calculations, e.g., higher level QM calculations on classical MD frames, or different force fields.^7^

## Supporting Information Available

Computational details of simulations, validation of binless DHAM results, calculation of reorganization energies (PDF)
