# Supplementary material for: Direct Calculation of Electron Transfer Rates with the Binless Dynamic Histogram Analysis Method
Source: J Phys Chem Lett. 2023 Oct 30;14(44):9935–42. doi: 10.1021/acs.jpclett.3c02624 (PMC10641885; doi:10.1021/acs.jpclett.3c02624)
Supplement: Supplementary file 1 — jz3c02624_si_001.pdf [file jz3c02624_si_001.pdf]

Direct Calculation of Electron Transfer Rates with the Binless Dynamic Histogram Analysis Method

Zsuzsanna Koczor-Benda,[1,2†] Teodora Mateeva,[3†] Edina Rosta[1*]

[1] Department of Physics and Astronomy, University College London, London, WC1E 6BT, United Kingdom

[2] The Department of Chemistry, University of Warwick, Coventry, CV4 7AL, United Kingdom

[3] Department of Physics, King's College London, London, WC2R 2LS, United Kingdom

[†] Equal contributions

[*] e-mail: e.rosta@ucl.ac.uk

S1. Alternative binless formulation

Instead of using the bias calculated for each data point as in Equation 4 of the manuscript, a binless formulation of DHAM can also be achieved by using the average (or median) bias $\bar{u}_i^l$ of all data points falling in bin *i* in simulation window *l* according to

$$M_{ji} = \sum_{k=1}^{N} \frac{T_{ji}^k}{\sum_{l=1}^{N} n_i^l \exp(-(\bar{u}_j^l - \bar{u}_i^l)/2k_B T}. \quad \text{(S1)}$$

We test this approach, which is analogous to that presented in Ref. [1] for the application of Ala5, on the ferrous-ferric ET example in Section S8.

S2. 1-D model potential

Monte Carlo (MC) simulations were carried out on an analytical model potential (details can be found in Ref.[2]) using 50 uniformly distributed umbrella windows in the range [0.05, 1.55], with K = 200 kcal/mol biasing spring constant for 5000 steps. The average of 20 repeated simulations was used to construct the free energy profile using 500 bins.

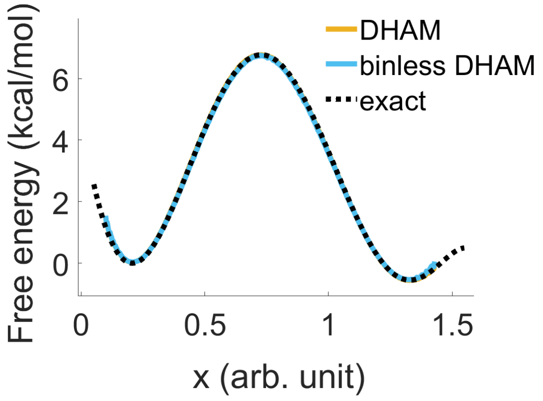

Figure S1. Free energy profiles for the 1-D model potential reconstructed with binless DHAM (blue) and DHAM (orange) compared to the exact profile (black dashed line).

S3. MD simulations for ferrous-ferric ET

All MD simulations in this work were performed with GROMACS version 2019.4 [3] and the Amber force field was used to model the systems [4]. The distance between the cations was fixed at 5.5 Å, the optimal separation of redox centers as determined in previous studies [5–7]. A cubic box with 567 water molecules and approximate size of 25 x 25 x 25 Å was used to solvate the system. The solvent was described with the TIP3P water model [8,9] and the LINCS constraint algorithm [10] was used for constraining bonded hydrogens. Minimization, equilibration and production steps were completed. The equilibration consisted of 500,000 steps using a step size of 1 fs. The production run consisted of 1,250,000 steps using a step size of 2 fs. Each frame of the production run was recorded and used in the analysis. The production step was completed in the constant-temperature, constant-volume ensemble (NVT). The temperature of 298 K was maintained with the Nose-Hoover thermostat. The Verlet cut-off scheme was employed to generate pair lists and the electrostatic interactions were evaluated with the Particle Mesh Ewald [11].

For the ET umbrella sampling calculations, charges were changed linearly in increments of 0.1 between reactants (Fe^{2+} + Fe^{3+}) and product (Fe^{3+} + Fe^{2+}), resulting in 11 independent simulations. The Van der Waals radius of the cations was also interpolated linearly. The potential energy was then re-evaluated for every window with every possible charge combination, resulting in 11 energy values for every MD frame, in total 1,250,000 frames for each umbrella window. The potential energy of each frame was re-evaluated using the rerun feature of mdrun.

S4. MD simulations for IET in (Q-TTF-Q)$^{-}$

A cubic box with 1112 water molecules and approximate size of 30 x 30 x 30 Å was used to solvate the system. The solvent was described with the TIP3P water model [8,9] and the LINCS constraint algorithm [10] was used for constraining bonded hydrogens. The equilibration step size was 1 fs for a total of 2,000,000 steps. The production run was completed with a step size of 2 fs for a total of 1,000,000 steps. The Nose-Hoover temperature coupling was used (303.15 K) with the Parrinello-Rahman pressure coupling for the production step. The Verlet cut-off scheme was employed to generate pair lists and the electrostatic interactions were evaluated with the Particle Mesh Ewald [11].

At the TS, the atomic charges of the two sides of the (Q-TTF-Q)$^{-}$ anion are symmetric (see Table 1), therefore, the same atom types can be used for each side. However, that is not the case for the reactant state, or the intermediate windows. For the first and for the intermediate windows, additional atom types were created. The charge of each atom at each window can be found in Table S1.

For the organic solvents tBOH, ETA and DCM the same protocol was used but this time the simulations were run longer to ensure the bulkier polar solvents were fully equilibrated. The production run was completed with a step size of 1 fs for a total of 40,000,000 steps. Only the last 10 ns were used to re-evaluate the potential energy, ensuring the respective system was fully equilibrated at this point. 30 x 30 x 30 Å cubic box was used to solvate the systems with the respective number of particles corresponding to the experimental density of each solvent (781 kg/m^3 for tBOH, 1322 kg/m^3 for DCM and 902 kg/m^3 for ethyl acetate).

Table S1. CHELPG atomic charges of the (Q-TTF-Q)$^{-}$ anion in the 4 simulation windows. The atom numbering is shown in the picture insert.

ATOM	WINDOW 1 (MINIMUM)	WINDOW 2	WINDOW 3	WINDOW 4 (TS)
C_1	-0.091513	-0.092180667	-0.092848333	-0.093516
C_2	-0.091513	-0.092180667	-0.092848333	-0.093516
C_3	-0.212622	-0.222454333	-0.232286667	-0.242119
C_4	-0.212622	-0.222454333	-0.232286667	-0.242119
H_5	0.157216	0.152515333	0.147814667	0.143114
H_6	0.157216	0.152515333	0.147814667	0.143114
C_7	0.001152	0.008055333	0.014958667	0.021862
C_8	0.042544	0.03565	0.028756	0.021862
C_9	-0.076267	-0.082016667	-0.087766333	-0.093516
C_{10}	-0.076267	-0.082016667	-0.087766333	-0.093516
C_{11}	-0.277497	-0.265704333	-0.253911667	-0.242119
C_{12}	-0.277497	-0.265704333	-0.253911667	-0.242119
H_{13}	0.132185	0.135828	0.139471	0.143114
H_{14}	0.132185	0.135828	0.139471	0.143114
C_{15}	0.660902	0.639588	0.618274	0.59696
O_{16}	-0.538238	-0.563756	-0.589274	-0.614792
C_{17}	0.660902	0.639588	0.618274	0.59696
O_{18}	-0.538238	-0.563756	-0.589274	-0.614792
C_{19}	0.518638	0.544745333	0.570852667	0.59696
O_{20}	-0.687509	-0.66327	-0.639031	-0.614792
C_{21}	0.518638	0.544745333	0.570852667	0.59696
O_{22}	-0.687509	-0.66327	-0.639031	-0.614792
S_{23}	-0.023899	-0.032792	-0.041685	-0.050578
S_{24}	-0.023899	-0.032792	-0.041685	-0.050578
S_{25}	-0.083244	-0.072355333	-0.061466667	-0.050578
S_{26}	-0.083244	-0.072355333	-0.061466667	-0.050578

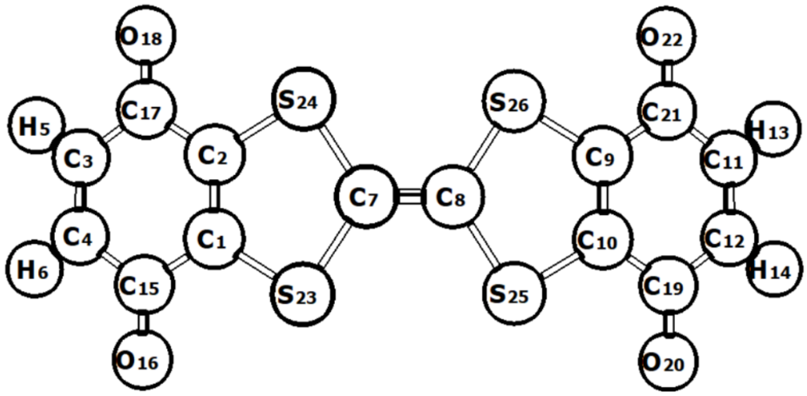

S5. Effects of changing the number of bins on free energy profiles

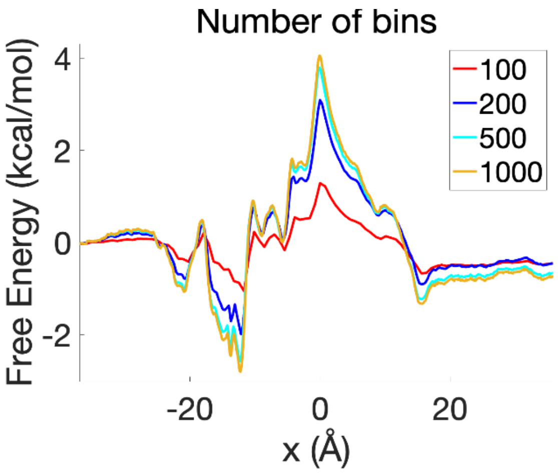

Figure S2. Binless DHAM free energy profiles with different number of bins for Na^+ passage through the GLIC ion channel.

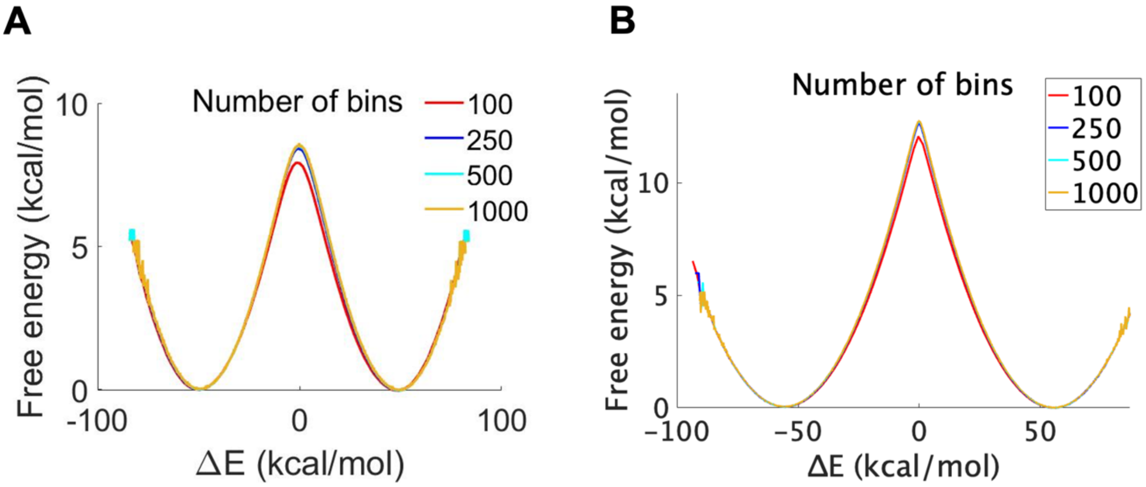

Figure S3. (A) Binless DHAM free energy profiles with different number of bins for ET in (Q-TTF-Q)$^{-}$, using 2 fs lag time and H_{ab} = 4.2 kcal/mol and (B) free energy profiles with different number of bins for ET in the ferrous-ferric system, using lag time 2 fs and H_{ab} = 0.2 kcal/mol.

S6. Average vs instantaneous bias values

Calculating the mean bias for all data points in bin *i* for each simulation window (according to Equation (S1)) instead of each data point (Equation (4) of manuscript) has a negligible effect on the results with lag time 2 fs. However, if the lag time is increased to 20 fs, the small number of observations of large energy gaps causes a noisy free energy profile, with the original formulation (Figure S4/A) giving numerically more stable results than the mean (Figure S4/B).

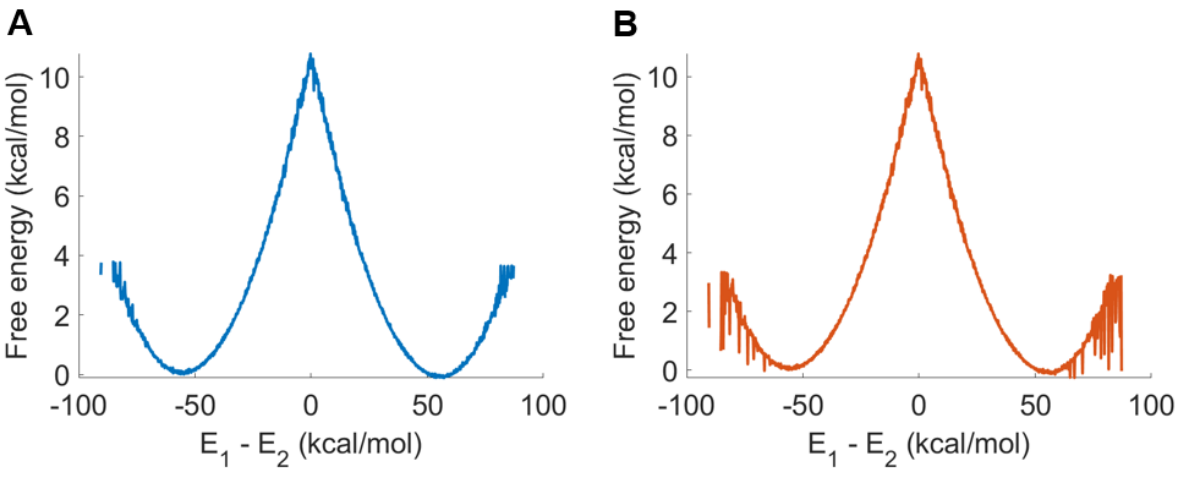

Figure S4. Binless DHAM profiles unbiased at the actual datapoints (blue) vs. the mean of all datapoints in the corresponding bin (red) for the ferrous-ferric ET. The lag time was increased to 20 fs to investigate the numerical performance of the approaches, while the number of bins was kept at 1000.

S7. Comparison of binless DHAM and MBAR profiles

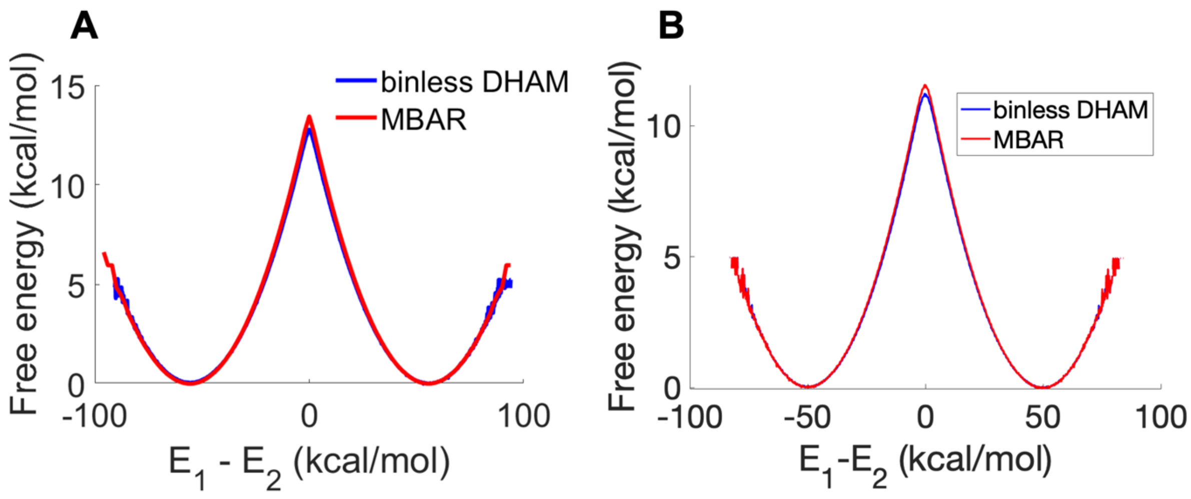

Figure S5. Binless DHAM (blue) and MBAR (red) free energy profiles for (A) ferrous-ferric ET and (B) ET in (Q-TTF-Q)$^{-}$ in water. For both reactions, 1000 bins and 2 fs lag time were used with binless DHAM, and 100 bins with MBAR. H_{ab} values of (A) 0.2 kcal/mol and (B) 0.97 kcal/mol have been used.

S8. Determining the reorganization energy from diabatic free energy profiles

To determine reorganization energy λ, quadratic functions are fitted to $G_{1,2}$ (Figure S6), and the free energy difference is taken between the reactant and product minimum structures for each curve. For ferrous-ferric ET (Figure S6/A), we get λ values of 53.0 and 53.2 kcal/mol, from state 1 and 2 respectively. For further calculations we use their average value, 53.1 kcal/mol. For IET in (Q-TTF-Q)$^{-}$ (Figure S6/B) we get λ = 48.4 kcal/mol from both curves.

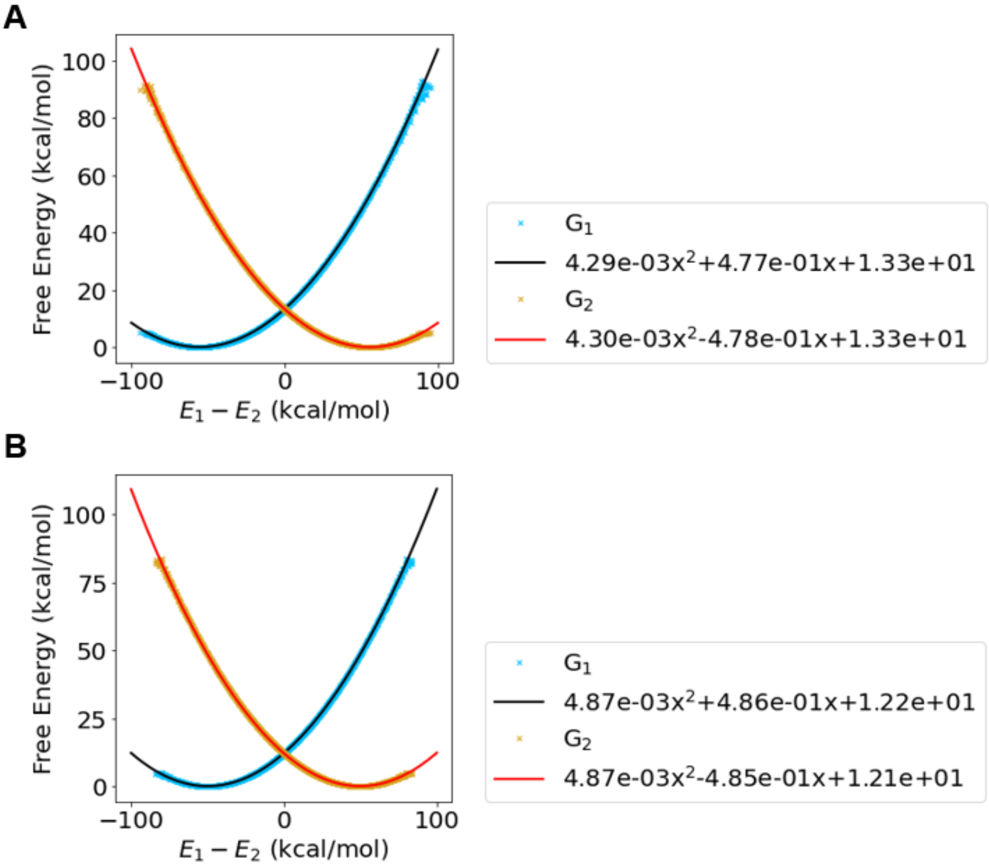

Figure S6. Binless DHAM free energy profiles for (A) ferrous-ferric ET and (B**)** IET in (Q-TTF-Q)$^{-}$ depicting diabatic states 1 (blue) and 2 (orange) as well as quadratic fits (black and red) on data between bins 40 and 960.

References

(1) Stelzl, L. S.; Kells, A.; Rosta, E.; Hummer, G. Dynamic Histogram Analysis To Determine Free Energies and Rates from Biased Simulations. *J Chem Theory Comput* **2017**, *13* (12). https://doi.org/10.1021/acs.jctc.7b00373.

(2) Rosta, E.; Hummer, G. Free Energies from Dynamic Weighted Histogram Analysis Using Unbiased Markov State Model. *J Chem Theory Comput* **2015**, *11* (1), 276–285. https://doi.org/10.1021/ct500719p.

(3) van der Spoel, D.; Lindahl, E.; Hess, B.; Groenhof, G.; Mark, A. E.; Berendsen, H. J. C. GROMACS: Fast, Flexible, and Free. *Journal of Computational Chemistry*. **2005**. https://doi.org/10.1002/jcc.20291.

(4) Wang, J.; Wolf, R. M.; Caldwell, J. W.; Kollman, P. A.; Case, D. A. Development and Testing of a General Amber Force Field. *J Comput Chem* **2004**, *25* (9). https://doi.org/10.1002/jcc.20035.

(5) Sit, P. H. L.; Cococcioni, M.; Marzari, N. Realistic Quantitative Descriptions of Electron Transfer Reactions: Diabatic Free-Energy Surfaces from First-Principles Molecular Dynamics. *Phys Rev Lett* **2006**, *97* (2). https://doi.org/10.1103/PhysRevLett.97.028303.

(6) Logan, J.; Newton, M. D. Ab Initio Study of Electronic Coupling in the Aqueous Fe 2+-Fe3+ Electron Exchange Process. *J Chem Phys* **1983**, *78* (6), 4086–4091. https://doi.org/10.1063/1.445136.

(7) Kuharski, R. A.; Bader, J. S.; Chandler, D.; Sprik, M.; Klein, M. L.; Impey, R. W. Molecular Model for Aqueous Ferrous-Ferric Electron Transfer. *J Chem Phys* **1988**, *89* (5), 3248–3257. https://doi.org/10.1063/1.454929.

(8) Jorgensen, W. L.; Chandrasekhar, J.; Madura, J. D.; Impey, R. W.; Klein, M. L. Comparison of Simple Potential Functions for Simulating Liquid Water. *J Chem Phys* **1983**, *79* (2). https://doi.org/10.1063/1.445869.

(9) Price, D. J.; Brooks, C. L. A Modified TIP3P Water Potential for Simulation with Ewald Summation. *Journal of Chemical Physics* **2004**, *121* (20). https://doi.org/10.1063/1.1808117.

(10) Hess, B.; Bekker, H.; Berendsen, H. J. C.; Fraaije, J. G. E. M. LINCS: A Linear Constraint Solver for Molecular Simulations. *J Comput Chem* **1997**, *18* (12). https://doi.org/10.1002/(SICI)1096-987X(199709)18:12<1463::AID-JCC4>3.0.CO;2-H.

(11) Darden, T.; York, D.; Pedersen, L. Particle Mesh Ewald: An N·log(N) Method for Ewald Sums in Large Systems. *J Chem Phys* **1993**, *98* (12). https://doi.org/10.1063/1.464397.
